# Novel machine learning approaches revolutionize protein knowledge

**DOI:** 10.1016/j.tibs.2022.11.001

**Published:** 2023-04

**Authors:** Nicola Bordin, Christian Dallago, Michael Heinzinger, Stephanie Kim, Maria Littmann, Clemens Rauer, Martin Steinegger, Burkhard Rost, Christine Orengo

**Affiliations:** 1 Institute of Structural and Molecular Biology, University College London, Gower St, WC1E 6BT London, UK; 2 Technical University of Munich (TUM) Department of Informatics, Bioinformatics and Computational Biology – i12, Boltzmannstr. 3, 85748 Garching/Munich, Germany; 3 VantAI, 151 W 42nd Street, New York, NY 10036, USA; 4 TUM Graduate School, Center of Doctoral Studies in Informatics and its Applications (CeDoSIA), Boltzmannstr. 11, 85748 Garching, Germany; 5 School of Biological Sciences, Seoul National University, Seoul, South Korea; 6 Artificial Intelligence Institute, Seoul National University, Seoul, South Korea; 7 Institute for Advanced Study (TUM-IAS), Lichtenbergstr. 2a, 85748 Garching/Munich, Germany; 8 TUM School of Life Sciences Weihenstephan (TUM-WZW), Alte Akademie 8, Freising, Germany

**Keywords:** protein structure prediction, machine learning, AI, pLM, structure alignment, embeddings, AlphaFold2

## Abstract

Breakthrough methods in machine learning (ML), protein structure prediction, and novel ultrafast structural aligners are revolutionizing structural biology. Obtaining accurate models of proteins and annotating their functions on a large scale is no longer limited by time and resources. The most recent method to be top ranked by the Critical Assessment of Structure Prediction (CASP) assessment, AlphaFold 2 (AF2), is capable of building structural models with an accuracy comparable to that of experimental structures. Annotations of 3D models are keeping pace with the deposition of the structures due to advancements in protein language models (pLMs) and structural aligners that help validate these transferred annotations. In this review we describe how recent developments in ML for protein science are making large-scale structural bioinformatics available to the general scientific community.

## Highlights

Two artificial intelligence (AI)-based methods for protein structure prediction, AlphaFold 2 and RoseTTAFold, increase dramatically the quality of structural modeling from sequence, nearing experimental accuracy.
Protein language models encode the written language of proteins, allowing for more accurate annotations and predictions than homology-based methods.
Most model organisms, neglected disease pathogens, and proteins with curated annotations have models available with varying quality, aiding wet-laboratory experiments targeting single-question issues.
Ultrafast alignment tools can traverse the protein space by both sequence and structure to identify remote evolutionary relations previously precluded to older and slower methods.
Preliminary analyses of predicted AlphaFold 2 3D-models from 21 model organisms suggest that the majority (>90%) of globular domains in proteins can be assigned to currently characterized domain evolutionary superfamilies.

## From protein sequence and structure to function through ML

The number of experimentally determined, high-resolution structures deposited in the Protein Data Banki (PDB) [1] has grown immensely since its beginning in 1976, enabling research into biological mechanisms, and in turn the development of novel therapeutics and industrial applications. This growth is, however, outpaced exponentially by that of known protein sequences increasingly impacted by high-throughput metagenomic experiments which yield billions of entries per experiment. Closing the ever-increasing gap between protein sequence and annotations of structure and function is thus a desideratum in molecular and medical biology research.

Most proteins comprise two or more structural domains [2], that is, constituents with compact structures assumed to fold largely independently. Structural domains are often associated with specific functional roles [3], although functional sites can be formed from multiple domains [3]. These structural domains – often dubbed ‘folds’ – recur in nature [4], and have been estimated to be limited to a number in the order of thousands [5]. Folds resemble more the Plato’s allegory of the cave: more the image or idea or concept than the real object (Plato Politeia [6]); this image helps to map relations between proteins.

Various resources emerged to classify domain structures in evolutionary families and fold groups (e.g., SCOPii [7], CATHiii [8], SCOPeiv [9], and ECODv [10]), and these have saturated at about 5000 structural families and about 1300 folds over the past decade, despite structural genomics initiatives targeting proteins likely to have new folds [11]. As increasingly powerful sequence profile methods [12., 13., 14.] have identified structural families in completely sequenced organisms (complete proteomes), studies suggest that up to 70% of all domains resemble those already classified in SCOP or CATH [3,15., 16., 17.]. Trivially, the distribution of family size follows some power law: most families/folds are small or species-specific, but a few hundred are very highly populated, tend to be universal across species, and have important functions [8]. In parallel, flexible or intrinsically disordered regions (IDRs) [18] making up 20–30% of all residues in a given proteome have been associated with protein function [19., 20., 21.]. As much as structural domains could be thought of as the structural units of proteins, the millions of domain combinations create the immense diversity of functional repertoires.

Since the details of function for most proteins in most organisms remain uncharacterized, understanding how domains evolve and combine to modify function would be a major step in our quest to understand and engineer biology. Protein structure data can provide a waymark, and exciting advances in structure prediction over the past year suggest that a landmark has been reached [22]. While structure prediction has steadily improved over time, thanks to the exponential growth in protein sequence data and covariation methods, this new era was kick-started by the remarkable performance of AlphaFoldvi (AF) at CASP13 [23]. The method, though, was not made available to the scientific community, resulting in various groups trying to replicate the features behind its breakthrough performance. Methods that were previously state-of-the-art released new versions based on these advancements, such as RoseTTAFold [24] and PREFMD [25]. DeepMind’s AF2 outperformed others in CASP14 [26], and reports suggest that high-quality models can be comparable to crystallographic structures, with competing methods reproducing DeepMind’s results [24,27,28]. DeepMind has recently announced the availability of 214 million putative protein structures for the whole of UniProt, which are available through the AF Database [29] and 3D-Beacons platform at the European Bioinformatics Institute (EBI). The latter provides AF models and other models by other prediction methods [30]. This 1000-fold increase in structural data requires equally transformative developments in methods for processing and analyzing the mix of experimental and putative structure/sequence data, including methods reliably predicting aspects of function from sequence alone [31., 32., 33.], and methods to quickly sift through putative structures [34].

In this review, we consider recent developments in deep learning, a branch of **ML** (see Glossary) operating on sequence and structure comparisons that enable highly sensitive detection of distant relationships between proteins. These will allow us to harness important insights on putative structure space, on domain combinations, and on the extent and role of disorder. One important observation from this review: no single modality has all the answers. Instead, protein sequence, **evolutionary information**, latent **embeddings** from **pLMs**, and structure information all play key roles in helping to uncover how proteins fold and act. Application of these tools have enabled rapid evolutionary classification of good quality AF2 models (defined as AF2 Predicted Local Distance Difference Test (pLDDT) ≥ 70 [22]) for 21 model organisms, including human (*Homo sapiens*), mouse (*Mus musculus*), rat (*Rattus norvegicus*), and thale cress (*Arabidopsis thaliana*) [35]. We review the insights derived from these studies and the future opportunities they bring for understanding the links between protein structural arrangements and their functions.

## Sequence-based approaches to find homologs

### Sequence similarity and evolutionary information provide gold-standard baselines

Comparing a query protein sequence against the growing sequence databases can reveal a goldmine of evolutionary information encoded in related (similar) sequences (Table 1). Closely related sequences with annotations of function and structure have successfully been used for **homology-based inference (HBI)**, that is, the transfer of annotations from labeled to sequence-similar yet unlabeled proteins [36,37]. Beyond annotation transfer, evolutionary information condensed in multiple sequence alignments (MSAs) can serve for *de novo* protein function and structure prediction methods, which have ranked highly for decades in independent evaluations [37., 38., 39., 40.]. However, the runtime and parameter sensitivity of popular solutions to generate MSAs [12,13,41], in combination with selection biased sequence datasets, creates major bottlenecks: (i) slow runtime, (ii) uninformative MSAs from inappropriate default parameters, from difficult to align families (e.g., IDRs), or from lack of diversity for understudied or species-specific families. Uninformative MSAs affect the prediction quality even for AF2 [22,42,43]. While advances in computer hardware coupled with clever engineering overcame some of the speed limitations [14,44], the faster-than-Moore’s-law [45] growth of sequence databases demands alternative or complementary solutions.

**Table 1:** Advantages and disadvantages of methods for homology detection

	Advantages	Disadvantages
Homology-based inference (HBI)	• Highly reliable • Interpretable	• Computationally expensive • Sensitive to choice of databases and parameters
Embedding-based annotation transfer (EAT)	• Fast inference, that is, generation of embeddings • Data-driven feature learning and extraction --> reduced human bias • Detection of distant homologs	• Computationally expensive pretraining (only has to be done once) • Choice of dataset, redundancy level, preprocessing is still human biased
Contrastive learning	• Specialization for specific use-case improves performance • Detection of distant homologs	• Pre-trained model is not generally applicable for identification of homologs for all aspects of protein function
Supervised learning	• Detection of distant homologs	• Difficult to extend to more classes • Requires enough data
Structure-based annotation transfer (SAT)	• Detection of very distant homologs • Highly interpretable since alignments can be interpreted visually using structure	• Computationally expensive • No standardized metrics of similarity [root mean square deviation (RMSD), TM-score returned by TM-align method]

### pLMs: deep learning learns protein grammar

**Figure 1:**
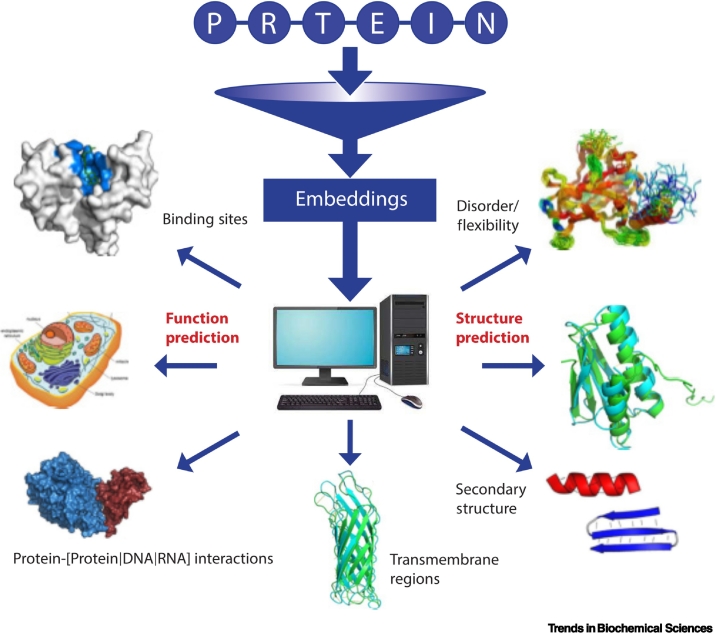
Overview of embeddings applications in protein structure and function characterization. Images were retrieved from Wikipedia (alpha helix and beta strand, binding sites), Creative Proteomics (cell structure), and bioRxiv (Transmembrane Regions - CASP13 Target T1008, Structure Prediction - PDB 1Q9F) with permission from the authors.

One alternative to direct evolutionary information extraction is leveraging deep learning teaching machines to encode information contained in billions of known protein sequences by adapting so-called language models (LMs) from natural language processing (NLP) to learn aspects of the ‘grammar’ of the language of life as encoded in protein sequences [46., 47., 48., 49., 50., 51.]. Where traditional ML models are trained to learn from labeled data (i.e., data with annotations) (supervised training), pLMs implicitly learn data attributes, such as constraints (evolutionary, structural, or functional) shaping protein sequences (dubbed self-supervised learning). This can be achieved either by autoregression, that is, training on predicting the next token (the word in text, the residue in pLMs) given all previous tokens in a sequence, or via masked-language modeling (i.e., by training on reconstructing corrupted sequences from noncorrupted sequence context) [47,48,50]. Repeating this on billions of sequences forces the pLM to learn properties and statistical commonalities of the underlying protein language. The resulting solutions can be transferred to other tasks (transfer learning) to predict many different phenotypes [43,52., 53., 54., 55.] (Figure 1).

Technically, this can be achieved by extracting the hidden states from the pLM referred to as embeddings. One key advantage of pLMs over evolutionary information is that the computation-heavy information extraction (learning the pLM) needs to be done only once during model training on efficient, high-performance computing centers. The extraction and use of the embeddings, by contrast, is done efficiently on consumer-grade hardware such as modern personal computers or even laptops.

### pLMs improve the prediction of protein function

Since the introduction of the first general-purpose pLMs around 3 years ago [46,48,50,56], pLMs have been shown to astutely capture aspects of protein structure, function, and evolution just from the information contained in databases of raw sequences [32,43,47,48,50,52,53,57,58]. In an analogy with HBI, which transfers annotations based on sequence similarity, **embedding-based annotation transfer (EAT)** captures more information through comparing proteins in embedding, not sequence space [59,60]. Without any domain-specific optimization, and without ever seeing any labeled data, simple EAT outperformed HBI by a large margin and ranked among the top ten methods for predicting the molecular function of a protein during Critical Assessment of Functional Annotation 4 (CAFA4) [31]. Adding domain optimizations, EAT predicted proteins according to the CATH classification [8,60] beyond what could be detected by advanced sequence profile methods [61]. The power of pLMs was confirmed as CATHe revealed distant evolutionary relationships, not detected by sequence profile methods, yet confirmed by structure comparison of AF2-predicted models.

### Leap in protein structure prediction combines ML with evolutionary information and hardware

Considered 2021’s method of the year [62], AF2 [22] combines advanced deep learning with evolutionary information from larger MSAs – obtained from the BFD with 2.1 billion sequences [63] or MGnifyvii [64] with 2.4 billion sequences, as opposed to UniProtviii with 231 million [65] – and more potent computer hardware to make major advances in protein structure prediction, providing good quality models for at least 50% of the likely globular domains in UniProt sequences. All top structure prediction methods, including AF2 and RoseTTAFold, rely on **evolutionary couplings (EV)** [66] extracted from MSAs. These approaches detect protein residues in close proximity and coevolving. The adequate preprocessing of this information has been advancing crucially over the past decades: for example, through direct coupling analysis sharpening this signal [67,68]. Although the leap of AF2 required this foundation in 2021, future advances may build their models on a different foundation [43].

### Protein structure proxies function for distant homologs

**Figure 2:**
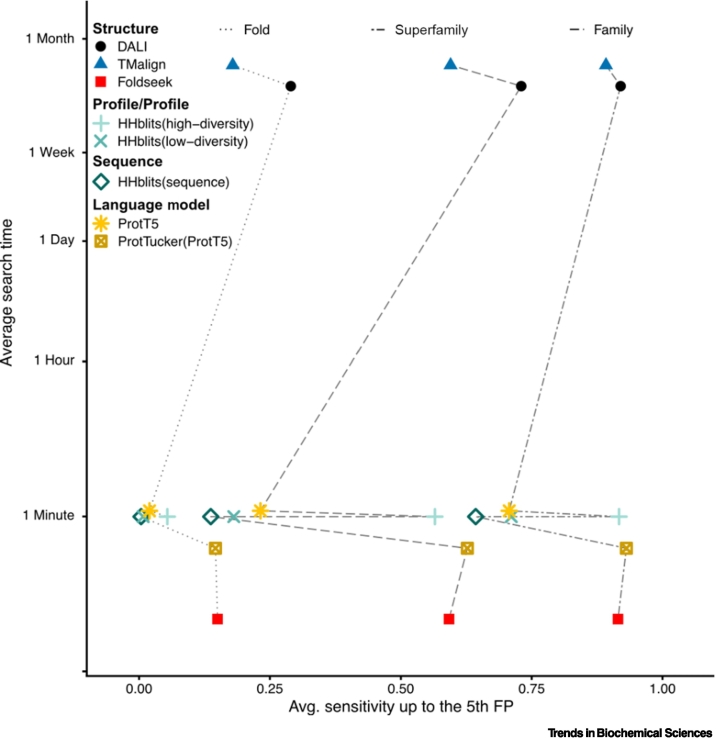
Comparison of search sensitivity and speed for language models, sequence/profile-profile and structure aligner. Average sensitivity up to the fifth false positive (x-axis) for family, superfamily, and fold measured on SCOP40e (version 2.01) [9] against average search time for a single query (y-axis) of 100 million proteins. Per SCOP40e domain we compute the fraction of detected true positives for family, superfamily, and fold up to the 5th false positive (FP) (= different fold), and plotted the average sensitivity over the domains (x-axis).

As alternatives to HBI or EAT, **structure-based annotation transfer (SAT)** emerged. SAT more reliably captures distantly related proteins (Figure 2). With the recent breakthrough in protein structure prediction [22] solving structures computationally at near-X-ray quality [26], new possibilities to apply SAT at the proteome scale have arisen. A large new collection of *in silico* predicted structures is available through the AF Protein Structure Database (AFDB)ix [35], which has been analyzed through fold recognition algorithms to refine protein families and to discover novel protein folds. For instance, by mining structures with a widely used structural alignment tool (DALI) [69], a new member of the perforin/gasdermin (GSDM) pore-forming family in humans was identified in spite of having only 1% sequence identity with the GSDM family [70,71]. Furthermore, the expanded search to all proteomes, covering 356 000 predicted structures, discovered 16 novel perforin-like proteins [72].

### Faster solutions for structure–structure alignments enable high-throughput analyses in seconds

Despite efforts to improve the speed and sensitivity of structural aligners, traditional approaches [69,73., 74., 75.] are too slow to cope with the rapidly increasing size of predicted structure databases [35,76] (Figure 2). Hence, novel ideas for structural comparison algorithms are emerging to accelerate run times. These methods gain in speed by representing structures in a compressed form (Table 2).

**Table 2:** Advantages and disadvantages of methods for structure-based homology detection

Approach	Tools	Advantages	Disadvantages	Representation	Similarity calculation	Alignment method
Structure fragments	Geometricus	Fast structure similarity search tool Accurate compared to other alignment-free techniques	Global comparison only Sensitivity is limited	Backbone encoded as fixed sized fragments as moment invariants	Vector distance similarity of Geometricus embedding vectors	Not available
	RUPEE	Fast structure database search toolEasy to use through webserver	Global comparison onlySensitivity is limited	Backbone encoded as fixed sized fragments of backbone torsion angles	Jaccard-similarity of torsion fragments or TM-score	TM-align [74]
Structure volume	BioZernike	Protein chain, and quaternary structure topology free technique (avoids chain matching problem)Both methods provide easy to use webserver	Global comparison onlySearches similar surface shape, which is not sensitive	3D Zernike descriptor of volume	Pretrained distance function to compare two volumes	Not available
	3D-AF-Surfer			3D Zernike descriptors of volume	Predicted probability of being in the same fold by neural network	CE [75]
Structural alphabets	Foldseek	Fast and accurate structure alignment toolLocal or global alignmentEasy to use through webserver	No quaternary structures comparison	3Di alphabet that describes tertiary residue–residue interactions	E-value, LDDT, TM-score	Structural Smith–Waterman or TM-align

One way to compress structural information is to break structures into fixed-size fragments. Geometricus [77] represents proteins as a bag of shape-mers: fixed-sized structural fragments described as moment invariants. It was used to cluster the AFDB and PDB using non-negative matrix factorization to identify novel groups of protein structures [78]. RUPEE [79] is another method that breaks structures into structural fragments. It discretizes protein structures by their backbone torsion angles and then compares the Jaccard similarity of bags of torsion fragments of the two structures. The top 8000 hits are then realigned by TM-align [74] in top-align mode.

Another category of tools represents tertiary structure as discretized volumes and compares these. BioZernike [80], for example, approximates volumes through 3D Zernike descriptors and compares these by a pretrained distance function. 3D-AF-Surfer [15] also applies 3D Zernike descriptors followed by a support vector machine (SVM) trained to calculate the probability of two structures being in the same fold. Results are ranked by the SVM scores, while individual hits can be realigned using combinatorial extension (CE) [75].

The fastest category of structural aligners represents structures as sequences of a discrete structural alphabet. Most of these alphabets discretize the backbone angles of the structure [81., 82., 83.], however at a loss of information in the structured regions. Another type of structural discretization to a sequence was proposed by Foldseekx [34]. It describes tertiary residue–residue interactions as a discrete alphabet. It locally aligns the structure sequences using the fast MMseqs2xi algorithm [84]. Foldseek achieves the sensitivity of a state-of-the-art structural aligner like TM-align, while being at least 20 000 times faster.

Sequence-based structural alignment tools are well equipped to handle the upcoming avalanche of predicted protein structures. Efficient storage of structure information and queries against these makes searches against hundreds of millions of structures feasible. Representing structures as sequences allows us to also adapt fast clustering algorithms like Linclust [84] to compare billions of structures within a day. We expect current tools to further increase in sensitivity to match or exceed the performance of DALI [69] (Figure 2).

**Figure 3:**
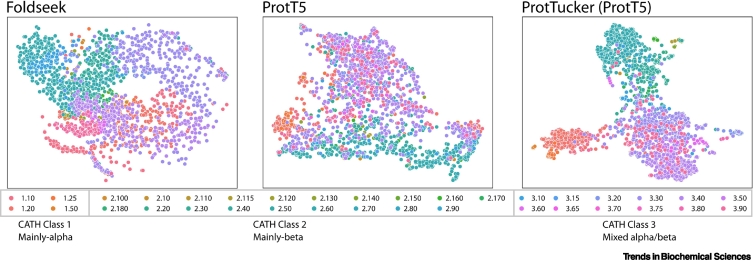
Visual analysis of the structure space spanned by CATH domains expanded by AlphaFold 2 (AF2) models. We showcase how distance in either structure (left) or embedding space (middle and right) can be used to gain insight into large sets of proteins. Simply put, we used pairwise distance between proteins to summarize ~850 000 protein domains in a single 2D plot and colored them according to their CATH class and architecture. This exemplifies a general-purpose tool for breaking down the complexity of large sets of proteins and allows, for example, detection of large-scale relationships that would otherwise be hard to find, or to detect outliers. More specifically, ~850 000 domains were structurally aligned using Foldseek [34] (left) in an all-versus-all fashion, resulting in a distance matrix based on the average pairwise bitscore within a superfamily as superfamily distances. The domain sequences were converted to embeddings using the ProtT5 (center) and ProtTucker (right) protein language models (pLMs). Similarly to the structural approach, the distance matrix between superfamilies were calculated using the average euclidean distance between embeddings belonging to different superfamilies. Using different modalities (i.e., structure and sequence embeddings) for computing distances on the same set of proteins, provides different, potentially orthogonal angles on the same problem which can be helpful during hypothesis generation. The resulting distance matrices were used as precomputed inputs for uniform manifold approximation and projection (UMAP) [121] and plotted with seaborn [122].

Embeddings from pLMs in combination with fast structural aligners (Foldseek) could be orthogonal in covering, classifying, and validating assignments in large swaths of protein fold space, as shown in Figure 3.

## Application of sequence and structure approaches to analyses of the protein universe

### Deep learning extends fold space

**Figure 4:**
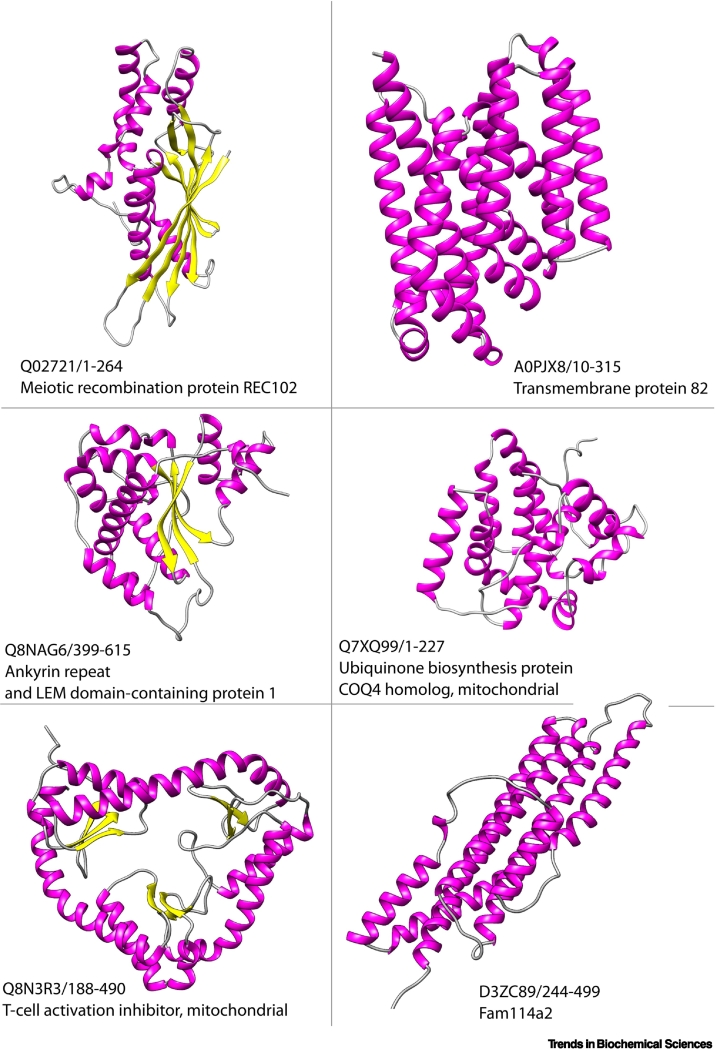
New folds in CATH-AlphaFold 2 (AF2). Examples of novel folds previously not encountered in CATH or Protein Data Bank (PDB). Structures are identified as novel folds if they have no significant structural similarity to domains or structures in the PDB using Foldseek as a comparison method. Each structure identifier is in the format UniProt_ID/start–stop with its current name in UniProt.

Following AFDB [35], structural analyses using fast, deep-learning-based methods (e.g., Geometricus, 3DZD) [15,77] suggested a slight predominance of mainly-alpha structures compared to the PDB, and predicted the existence of hundreds to thousands more structural families in the dataset [15]. About 75% of the AF2 structures are of sufficient global quality (pLDDT scores of ≥70) for these studies, depending on the analyses. However, even in these well-predicted 3D models, at least 26% of residues were of low model quality [16]. Recent studies showed that nearly 6% of these low-quality residues are predicted to be disordered by sequence-based approaches [78,85]. It is also clear that AF2 struggles to predict domains from small, species-specific families [16], suggesting that covariation data are needed for good-quality models. Preliminary analyses [16] revealed some very unusual structural architectures in which common folds are connected by large unordered regions or combined in quite regular arrangements using helical scaffolds (see also Figure 4).

We recently analyzed the proportion of predicted AF2 structural domains in the 21 model organisms that could be assigned to known superfamilies in CATH [16]. Only good-quality models were analyzed according to a range of criteria (pLDDT ≥70, large proportions of ordered residues, characteristic packing of secondary structure). We used well-established hidden Markov model (HMM)-based protocols and a novel deep-learning method (CATHe [61] based on ProtT5 [47]) to detect domain regions in the AF2 models, and Foldseek comparisons gave rapid confirmation of matches to CATH relatives [34]. We found that 92%, on average, of domains could be mapped to 3253 CATH superfamilies (out of 5600). We see that the proportion of residues in compact globular domains varies according to the organism, with well-studied model organisms having higher proportions of residues assigned to globular regions (ranging from 32% for *Leishmania infantum* to 76% for *Escherichia coli*).

By classifying good-quality AF2 models into CATH, we can expand the number of structurally characterized domains by ~67%, and our knowledge of fold groups in superfamilies (structurally similar relatives which can be well superposed) increases by ~36% to 14 859 [16]. As with other recent studies of AF2 models [15], we observe the greatest expansion in global fold groups for mainly-alpha proteins (2.5-fold). Less than 5% of CATH superfamilies (~250) are highly populated, accounting for 57.7% of all domains [8], and in these so called MEGAfamilies AF2 considerably increases the structural diversity, with some superfamilies now identified as having more than 1000 different fold groups, suggesting considerable structural plasticity outside the common structural core.

Our analyses identified 2367 putative novel families [16]. However, detailed manual analyses of 618 human AF2 structures revealed problematic features in the models, and some very distant homologies, with only 25 superfamilies verified as novel, suggesting that the majority of domain superfamilies may already be known. It is even likely that, as we bring more relatives into the AF2 superfamilies, links between current CATH superfamilies will be established and the number of superfamilies reduced. Indeed, most of the 25 new superfamilies identified possess domain structures with very similar architectures to those in existing CATH superfamilies; mainly-alpha structures (both orthogonal and up–down bundles) were particularly common, as were small alpha–beta two-layer sandwiches and mainly-beta barrels.

### Biological discoveries enabled by AF2 data

The availability of off-the-shelf solutions based on AF2 – both as a tool (ColabFoldxii [42], AF2 [22], AF-Multimer [86]) and as a collection of precomputed models (AFDB [35]) – is akin to the introduction of next-generation sequencing in small research groups enabled by nanopore sequencing. Suddenly, almost every protein of interest in various projects from medical to environmental research is not held back by a lack of experimentally derived structures in the PDB.

### Caveats

Although AF2 solves many challenging issues in structural modeling, its limitations have been rapidly identified by the community. All models have accompanying scores for each residue, indicating several aspects about the confidence of the prediction. For example, pLDDT gives the confidence for a particular residue, and predicted align error reflects inter-residue distances and local structural environments. Other measures are also provided. Models scoring below an average pLDDT value of 70 and containing large portions with incorrectly oriented secondary structure segments are unsuitable for most biological applications and do not reach the quality of experimentally derived structures [22]. Most issues could be related to the nature of the MSA the model is built upon, as shallowness or gaps in the alignment often result in a poor model [22,87].

Furthermore, overrepresentation of proteins with a particular folding state results in a model that is not representative of other alternative states [88]. Some models with low pLDDT point to IDRs that undergo disorder-to-order transition upon binding or are prone to fold-switching [89., 90., 91.]. Other features that may be available for experimental structures are missing from AF2 models, such as ions, cofactors, ligands, and post-translational modifications (PTMs) [92].

While some effects of sequence variants are captured by AF2, others – in particular point mutations or single amino acid variants – remain elusive to AF2, partly because predictions constitute a family-averaged more than a sequence-specific solution due to the MSA underlying each prediction [78,93].

### Small- and medium-scale applications of AF2

With these caveats and limitations taken into account, AF2 enabled both small- and large-scale applications to biological questions. The sudden availability of a reliable model relieved many research groups from long-term structural characterization efforts, allowing for targeted answers in conformational studies [94,95], oligomerization prediction [96,97], drug channel conformations [98], and early-stage assembly of complexes in disease [99]. Predictions derived from AF2 models helped in validating experimentally derived structures and complexes [100], aiding in solving X-ray crystallography for molecular replacement experiments [101], as well as replacing experimental characterization entirely when it fails with particularly tough cases [98]. Transmembrane proteins, in particular, are not easily solved by X-ray crystallography, so AF2 in combination with other techniques such as NMR are being used as an orthogonal validation for experiments where particular conformations of import channels were unclear [98].

### Large-scale applications of AF2

Large-scale applications of AF2 and AF-Multimer are creating entirely novel resources (AFDB [35]), complementing or expanding already established ones (CATH [8], APPRIS [102], and Membranome [103]), or enabling more focused collections and analyses, such as the characterization of the ‘metallome’ by identifying all metal-binding sites across proteomes [104], or shining light on the human dark proteome [105], or improving genomic annotation of the human genome through comparison of the predicted structures of 140k isoforms [106]. Since AF has now also released models for neglected tropical diseases, this will progress research on these often underfunded or ignored diseases. The recent release of protein structure models for the whole of UniProt will also enable large-scale analyses across the Tree of Life such as evolutionary studies on domain archaeology, among others.

### Unlocking new deep-learning venues

Thanks to the increase in high-quality structure predictions spawned by AF2, there is an increasing need to readily leverage 3D information by prediction methods. Instead of using representations that first map 3D structures to 2D (e.g., contact maps) or 1D (e.g., secondary structure) before feeding them to a predictor, such networks directly operate on 3D representations of macromolecules to make predictions about their properties. Using so-called ‘inductive bias’ when designing a network (i.e., incorporating domain knowledge directly into the architecture) avoids information loss during abstraction and enables the network to directly learn useful information from the raw 3D data itself. Recently, geometric deep-learning research [107], which focuses on methods handling complex representations like graphs, has seen a steady increase in adoption, accuracy, and potential opportunities [108]. Protein 3D structures are naturally fit for geometric deep-learning approaches, whether for supervised tasks, like the prediction of molecule binding [109], or unsupervised learning approaches, which could generate alternatives to learned representations from pLMs [110,111]. Geometric deep-learning approaches stand to benefit the most from large putative 3D structures sets, potentially unlocking further opportunities for alternative, unsupervised protein representations derived from structure, or deep-learning-based potentials to substitute expensive molecular dynamics simulations for molecular docking.

### A leveling effect

AF2 will help in eliminating an underlying bias in structural biology that has tended to focus more on drug discovery for human diseases, model organisms, or structures involved in pathogens. With its cheap footprint and cost, compared to traditional experimental means to characterize protein 3D structure, AF2 is neither constrained by access to expensive experimental instruments, nor to beam-time usually prioritized for large consortia. This will enable groups across the world to work on their proteins of interest without geographical or economical limitations. In a similar fashion to nanopore sequencing, model building could be done in real time for issues that are time- or location-sensitive (i.e., emerging pandemics, neglected tropical diseases), or limited to an individual, potentially allowing to transition from whole-genome sequencing to whole-proteome modeling, drug binding, and efficacy profiling.

## Concluding remarks and future perspectives

Computationally predicting protein properties with increasing accuracy using deep learning [22,53,57,58] remains crucial to build structures and assemblies that assist researchers in uncovering cellular machinery. Conveniently, the better these predictors become, the more interesting it is to hijack them to design new proteins that perform desired functions [112]. Recently, deep-learning approaches emerged that ‘hallucinate’ new proteins [113] which systems like AF2 confirm may fold into plausible structures. These tools can generate new protein sequences from start to finish or, similarly to text autocompletion, conditioned on part of a given sequence input [112], all within milliseconds of runtime. Coupled with blazingly fast predictors [43,114,115], millions of potentially foldable, ML-generated sequences can be screened reliably *in silico*, saving energy, time, and resources, requiring *in vitro* and *in vivo* experiments only at the most exciting stages of the experimental discovery process. Whilst not all designs fold, and caution is needed, an approach similar to spell correction in NLP but trained on millions of protein sequences allowed researchers to evolve and optimize existing antibodies to better perform a desired activity [116]. Additionally, approaches that generate protein sequences from 3D structure (in some sense, the opposite direction of the classical folding problem) will get more and more important in the post-AF2 era [117]. By selecting for sequence diversity conditioning on structure, new candidates for families may be found.

With booming putative structure databases, we see the emergence of analytical approaches leveraging a model similar to how UniProt’s mix of curated (SwissProt) and putative (TrEMBL) [65] sequence databases are being used. In part, we can build on years of advances in maintaining and searching sequence databases (e.g., to extract evolutionary relationships) to create tools to analyze structure databases instead, with performant tools already available [34]. However, mainstreaming structural analysis on billions of entries will require domain-specific infrastructure and tooling. Geometric deep learning may also assist this modality by bringing new unsupervised solutions similar to pLMs but trained on protein 3D structures instead [110]. With reliable solutions in this space, we expect practitioners to combine sequence and structural analysis.

Within the realm of ‘traditional’ pLMs, that solely learn information from large unlabeled protein sequence databases, there is still room for improvement, as highlighted by recent advances in NLP. For example, there are approaches optimizing the efficiency of LMs, especially, on long sequences either by modifying the existing attention mechanism [118] or by proposing a completely different solution not relying on the de facto standard (attention) [119]. Orthogonal to such architectural improvements, recent research highlights the importance of hyperparameter optimization [120] which goes away from the constant increase in model size and rather suggests to train ‘smaller’ models (still, those models have billions of parameters) on more samples. Taken together these improvements hold the potential to improve today’s sequence-based pLMs further.

Ultimately, we see that a plethora of effective and efficient ML tools operating on different modalities, each with unique strengths and weaknesses, become available at researchers’ fingertips. Further developments in structure- and sequence-based approaches are inevitably needed (see Outstanding questions), yet, even today, combining different ML and software solutions will bring researchers to an untapped world of novel mechanisms that await discovery.

Outstanding questions The best structural models rely on quality and availability of protein structures found in nature. How can we further improve these deep-learning methods without relying on previous structural knowledge?

How much structural novelty is hidden in metagenomes? Are we close to discovering all ways nature can shape a protein?

Could protein modeling entirely replace experimentally derived structures?

How can we use these methods to engineer a highly efficient enzyme function never encountered in living organisms?

Speed, accuracy, and coverage of structure predictions and methods are skyrocketing. How can these tools be improved to better probe the dark universe of uncharacterized proteins?

Could future methods build complexes of protein–protein interactions and models quickly and accurately enough to be used for precision medicine?

Can we use embeddings-based methods to predict evolution in sequences?

Can today’s deep-learning structure-prediction method capture dynamic changes in structure?

Alt-text: Outstanding questions

## Glossary

**Embedding-based annotation transfer (EAT)**: embedding-based annotation transfer applies the same logic as HBI but replaces sequence similarity (SIM) with similarity of embedding vectors (generalized sequences).
**Evolutionary couplings (EV)**: pairs of residues coupled through coevolution. The adequate preprocessing of these signals (e.g., through direct coupling analysis) was one important milestone toward AF2.
**Evolutionary information**: information compiled through the comparison of protein sequences and structures by grouping proteins into families connected through evolution. Typically, evolutionary information is compiled in so-called family profiles, or position-specific scoring matrices (PSSMs). The combination of evolutionary information and ML affected most breakthrough steps in protein structure prediction from 1992 to 2021.
**Homology-based inference (HBI)**: inference at the base of most protein annotations. Assume a query Q without known phenotype, and a protein K with experimentally known phenotype P, then HBI operates as follows: if the sequence similarity between Q and K exceeds some threshold T, we assume Q to have the same phenotype as K: if SIM(Q,K) > T -> phenotype (Q) = phenotype (K) = P. How to measure SIM and what value to choose for T depends on the type of phenotype and has to be empirically determined for each type of ‘phenotype’: for example, for 3D structure, and also for secondary structure, or for molecular function in the GeneOntology and also for binding particular classes of ligands. Incidentally, the evolutionary link in the word ‘homology’ is crucial, because protein design can generate protein pairs with similar sequences and dissimilar phenotypes.
**Machine learning (ML)**: computational systems that aim to emulate human intelligence, usually by means of statistics and probability.
**Protein language models (pLMs) and embeddings**: while language models (LMs) from natural language processing (NLP) understand natural language from data, pLMs aim at understanding the language of life through implicitly capturing the evolutionary, functional, and structural constraints on protein sequences. Effectively, such constraints can be learned from large sets of unannotated protein sequences, because all sequences that can ever be observed are not a random subset of all possible sequences. These constraints are captured in the connections of the ‘neurons’ used to train the pLMs, and can be written as vectors (rows of real-valued numbers) that are referred to as ‘the embeddings’.
**Structure-based annotation transfer (SAT)**: implementing a logic similar to HBI, SAT expands beyond the evolutionary connection. Instead, the assumption is that similar shapes (3D coordinates) and feature descriptors (e.g., density of charged residues in surface patch) affect some similarity in terms of other function-related phenotypes.

## Resources

^i^ www.wwpdb.org/

^ii^ https://scop2.mrc-lmb.cam.ac.uk/

^iii^ www.cathdb.info/

^iv^ https://scop.berkeley.edu/

^v^ http://prodata.swmed.edu/ecod/

^vi^ www.deepmind.com/blog/putting-the-power-of-alphafold-into-the-worlds-hands

^vii^ www.ebi.ac.uk/metagenomics/

^viii^ www.uniprot.org/

^ix^ www.alphafold.ebi.ac.uk/

^x^ https://search.foldseek.com/

^xi^ https://github.com/soedinglab/MMseqs2

^xii^ https://github.com/sokrypton/ColabFold
